# Melatonin and Sirtuins in Buccal Epithelium: Potential Biomarkers of Aging and Age-Related Pathologies

**DOI:** 10.3390/ijms21218134

**Published:** 2020-10-30

**Authors:** Annalucia Carbone, Natalia Linkova, Victoria Polyakova, Ekaterina Mironova, Ulduz Hashimova, Ahmed Gadzhiev, Khatira Safikhanova, Tatiana Kvetnaia, Julia Krylova, Roberto Tarquini, Gianluigi Mazzoccoli, Igor Kvetnoy

**Affiliations:** 1 Department of Medical Sciences, Division of Internal Medicine and Chronobiology Laboratory, Fondazione IRCCS Casa Sollievo della Sofferenza, 71013 San Giovanni Rotondo, Italy; 2 Department of Biogerontology, Saint Petersburg Institute of Bioregulation and Gerontology, 197110 Saint Petersburg, Russia; miayy@yandex.ru (N.L.); katrine1994@mail.ru (E.M.); kvetnaia@gerontology.ru (T.K.); 3 Department of Therapy, Geriatrics, and Anti-Aging Medicine, Academy of Postgraduate Education under FSBU FSCC of FMBA of Russia, 125310 Moscow, Russia; 4 Laboratory of Cell Biology and Pathology, Saint Petersburg State Pediatric Medical University, 194100 Saint Petersburg, Russia; vopol@yandex.ru; 5 Department of Physiology and Department of Pathology, Saint Petersburg State University, 199034 Saint Petersburg, Russia; igor.kvetnoy@yandex.ru; 6 Garayev Institute of Physiology, Azerbaijan National Academy of Sciences, Baku AZ1100, Azerbaijan; ulduz27@yahoo.com (U.H.); ahmed.hajiyev@yandex.com (A.G.); khatira.safikhanova.74@mail.ru (K.S.); 7 Department of Pathology, Pavlov First Saint-Petersburg State Medical University, 197022 Saint Petersburg, Russia; emerald2008@mail.ru; 8 Department of Clinical and Experimental Medicine, University of Florence, 50134 Florence, Italy; roberto.tarquini@uslcentro.toscana.it; 9 Inter-institutional Department for Continuity of Care of Empoli, University of Florence, 50134 Florence, Italy; 10 Center of Molecular Biomedicine, Saint-Petersburg Research Institute of Phthisiopulmonology, 191036 Saint Petersburg, Russia

**Keywords:** melatonin, sirtuins, buccal epithelium, aging, arterial hypertension

## Abstract

Melatonin (MT) and sirtuins (SIRT) are geroprotective molecules that hold back the aging process and the development of age-related diseases, including cardiovascular pathologies. Buccal epithelium (BE) sampling is a non-invasive procedure, yielding highly informative material for evaluating the expression of genes and proteins as well as the synthesis of molecules. Among these, MT and SIRTs are valuable markers of the aging process and age-related pathologies. The purpose of this study was to examine age-related expression patterns of these signaling molecules, in particular MT, SIRT1, SIRT3, and SIRT6 in BE of subjects of different ages with and without arterial hypertension (AH). We used real-time polymerase chain reaction (RT-PCR) and immunofluorescence analysis by confocal microscopy. We found that MT immunofluorescence intensity in BE decreases with aging, more evidently in AH patients. SIRT3 and SIRT6 genes expression and immunofluorescence intensity in BE was decreased in aging controls. In AH patients, SIRT1, SIRT3, and SIRT6 gene expression and immunofluorescence intensity in BE was decreased in relation to age and in comparison with age-matched controls. In conclusion, the evaluation of MT and sirtuins in BE could provide a non-invasive method for appraising the aging process, also when accompanied by AH.

## 1. Introduction

Aging and age-related diseases have been found to be associated with decreased synthesis of pineal and extrapineal melatonin (MT) [1,2,3,4]. MT regulates circadian rhythms of cells, organs, and tissues, as well as functions of the immune, cardiovascular, antioxidant systems, among others [5,6]. Cardiovascular pathology in general and arterial hypertension (AH) in particular are considered as age-related diseases, and MT has shown to be effective for the treatment of AH patients [7]. AH results in cellular metabolism disorders, particularly in mitochondrial dysfunction. MT exhibits an antioxidant effect and can normalize mitochondrial function. One of the links in the chain of AH pathogenesis is the imbalance in the renin-angiotensin system, whose functions are also supported by MT [8]. It is supposed that some anti-inflammatory, anti-oxidant, and other effects of MT cannot be fully appreciated due to a concomitant decrease in the synthesis of sirtuins, in particular SIRT1 [9].

Sirtuins (SIRT1–7) are members of the silent information regulator 2 (Sir2) family found in almost all species from bacteria to mammals and represent evolutionarily conserved nicotinamide-adenine-dinucleotide (NAD)-dependent class III histone deacetylases. SIRTs regulate transcription and cellular aging through the deacetylation of histone and non-histone target proteins [10,11,12]. SIRTs are involved in the regulation of metabolic pathways and the epigenetic regulation of gene expression in all living organisms. Mammalian SIRT1,2,6,7 are located in the nucleus, SIRT1,2 in the cytoplasm, and SIRT3,4,5 in mitochondria [13].

SIRT1 is involved in the regulation of inflammatory reactions and mitochondrial function, preventing tissue hypoxia [14]. A decrease in the synthesis of SIRT1 and SIRT6 is associated with aging of endothelial cells, development of atherosclerotic plaques, and myocardial and vascular pathology [15]. The vasoprotective properties of SIRT1, SIRT3, and SIRT6, which are evident in endothelial aging and AH, suggest a promising role in slowing down the aging process and point to these signaling molecules as potential tools for therapeutic strategies [16,17,18,19,20].

In view of the above, the purpose of this study was to investigate by means of real-time polymerase chain reaction (RT-PCR) and immunofluorescence microscopy the patterns of expression of SIRT1, SIRT3, and SIRT6 genes and encoded proteins and MT appearance in the buccal epithelium (BE) of subjects of different ages with and without AH.

## 2. Results

### 2.1. Expression of SIRT1, SIRT3, and SIRT6 mRNA in BE of Subjects of Different Ages with and without AH

SIRT1 mRNA expression in BE did not statistically differ between control subjects of different ages. In young/middle-aged, elderly, and long-lived AH patients, SIRT1 mRNA expression in BE was 1.67, 1.8, and 1.67 times lower as compared with age-matched controls (Table 1).

SIRT3 mRNA expression in BE in elderly and long-lived controls was 1.91 and 1.8 times lower as compared with young/middle-aged controls. SIRT3 mRNA expression in BE in young/middle-aged, elderly, and long-lived AH patients was 3.1, 1.59, and 1.32 times lower as compared with age-matched controls (Table 1).

SIRT6 mRNA expression in BE in elderly and long-lived controls was 2.27 and 2.13 times lower as compared with that in young/middle-aged controls. SIRT6 mRNA expression in BE in young/middle-aged, elderly, and long-lived AH patients was 2.27, 3.26, and 2.74 times lower as compared with that in age-matched controls. Considering AH patients, SIRT6 mRNA expression in BE in elderly and long-lived patients was 2.9 and 2.58 times lower as compared with that in young/middle-aged patients (Table 1).

### 2.2. Melatonin, SIRT1, SIRT3, and SIRT6 Immunofluorescence Intensity in BE in Subjects of Different Ages with and without AH

Immunofluorescence intensity of MT in BE in elderly controls was 1.9 times lower as compared with that in young/middle-aged subjects and was 2.8 times lower in long-lived subjects as compared with elderly controls.

**Figure 1:**
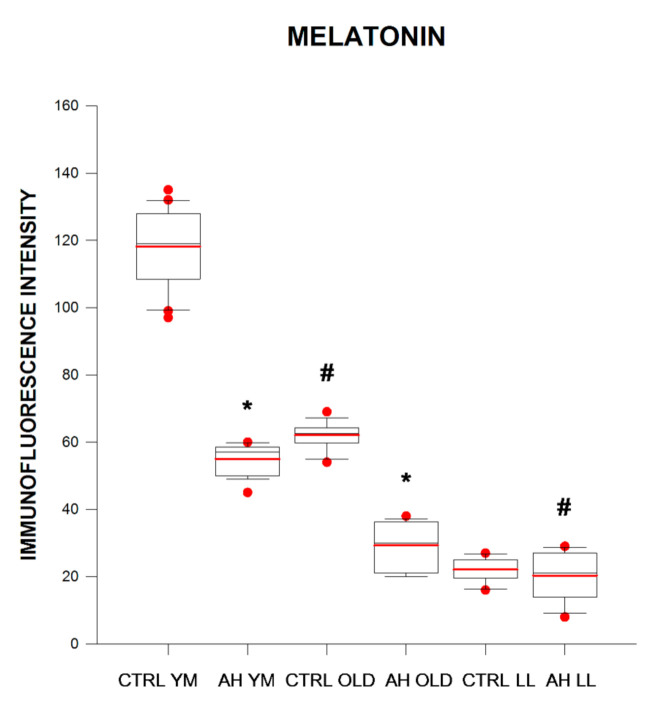
Immunofluorescence intensity (arbitrary units) of melatonin in buccal epithelium in healthy subjects (CTRL) and in patients with arterial hypertension (AH) of different ages (young–middle-aged YM, elderly OLD, and long-lived LL). * *p* < 0.05 as compared with the respective age-matched controls for patients with AH, **#** *p* < 0.05 as compared with the other age groups for controls and patients with AH. Box and whiskers show minimum, Q1, median (black line), mean (red line), Q3, maximum, outliers (circle).

**Figure 2:**
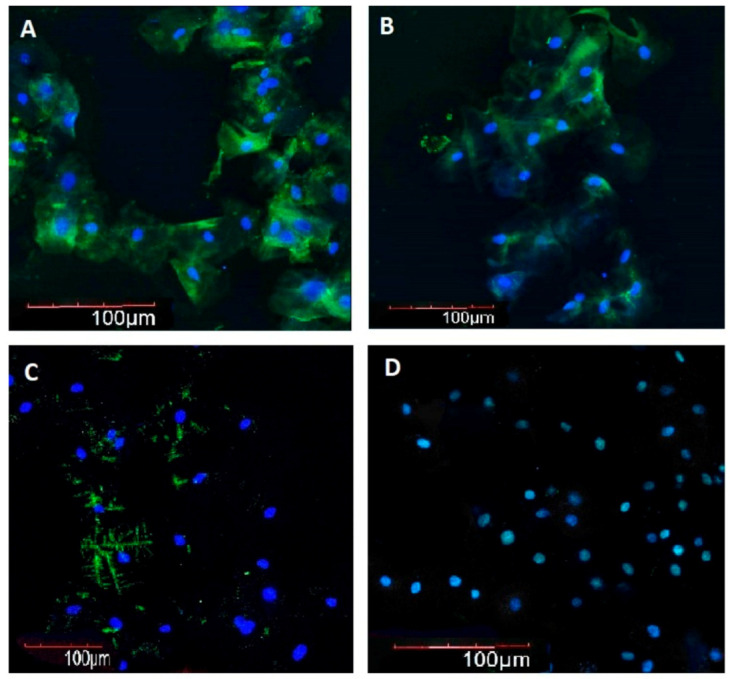
Immunofluorescence intensity of melatonin in buccal epithelium. 200×. Immunofluorescence staining with antibodies to melatonin (Alexa Fluor 488—green fluorescence). Nuclei were counterstained with Hoechst 33258—blue fluorescence. (**A**) Woman, 28 years old, control, (**B**) woman, 39 years old, arterial hypertension, (**C**) woman, 68 years old, control, (**D**) woman, 67 years old, arterial hypertension.

In young/middle-aged and elderly AH patients MT immunofluorescence intensity in BE was 2.2 and 2.1 times lower as compared with that in age-matched controls, respectively, whereas in elderly and long-lived AH patients, it was 1.9 and 2.8 times lower as compared with that in young/middle-aged AH patients, respectively (Figure 1 and Figure 2).

**Figure 3:**
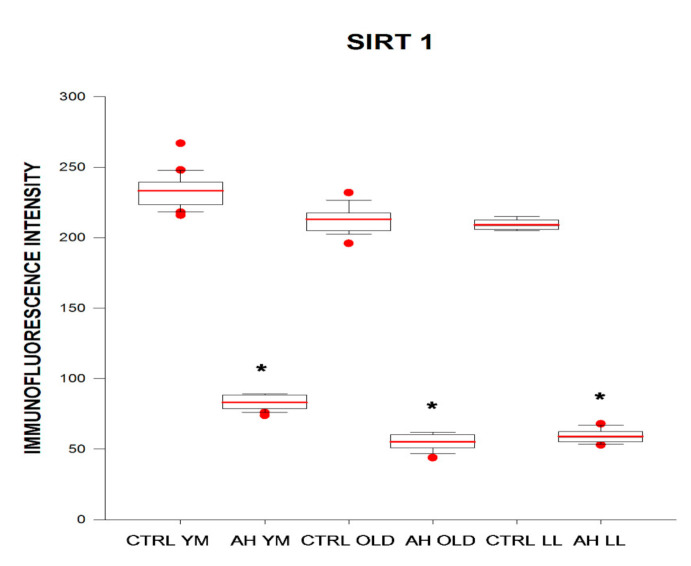
Immunofluorescence intensity (arbitrary units) of SIRT1 in buccal epithelium in healthy subjects (CTRL) and in patients with arterial hypertension (AH) of different ages (young–middle-aged YM, elderly OLD, and long-lived LL). * *p* < 0.05 as compared with the respective age-matched controls for patients with AH. Box and whiskers show minimum, Q1, median (black line), mean (red line), Q3, maximum, outliers (circle).

**Figure 4:**
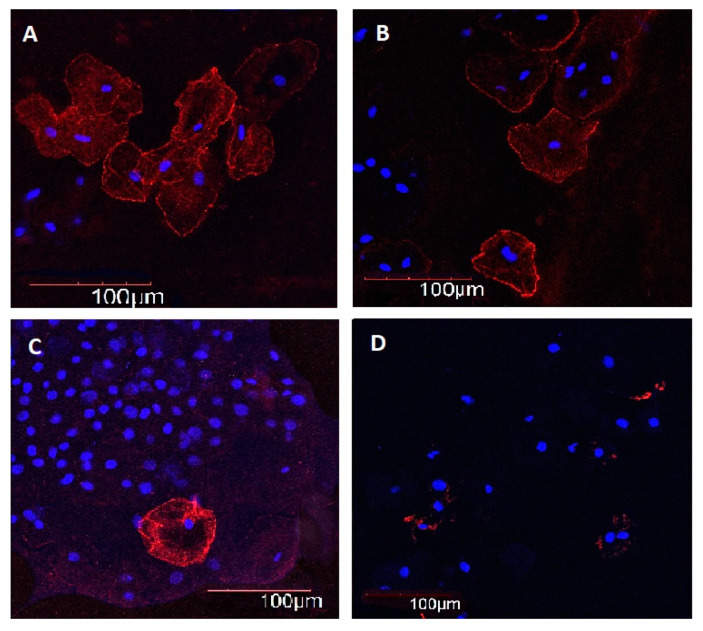
Immunofluorescence intensity of SIRT1 in buccal epithelium. 200×. Immunofluorescence staining with antibodies to SIRT1 (Alexa Fluor 567—red fluorescence). Nuclei were counterstained with Hoechst 33258—blue fluorescence. (**A**) Man, 43 years old, healthy, (**B**) man, 68 years old, healthy, (**C**) man, 91 years old, healthy, (**D**) woman, 39 years old, arterial hypertension.

Immunofluorescence intensity of SIRT1 in BE of control subjects did not differ among the age groups. In young–middle-aged, elderly, and long-lived subjects with AH, immunofluorescence intensity of SIRT1 in BE was 2.8, 3.9, and 3.5 times lower as compared with age-matched controls, respectively (Figure 3 and Figure 4).

**Figure 5:**
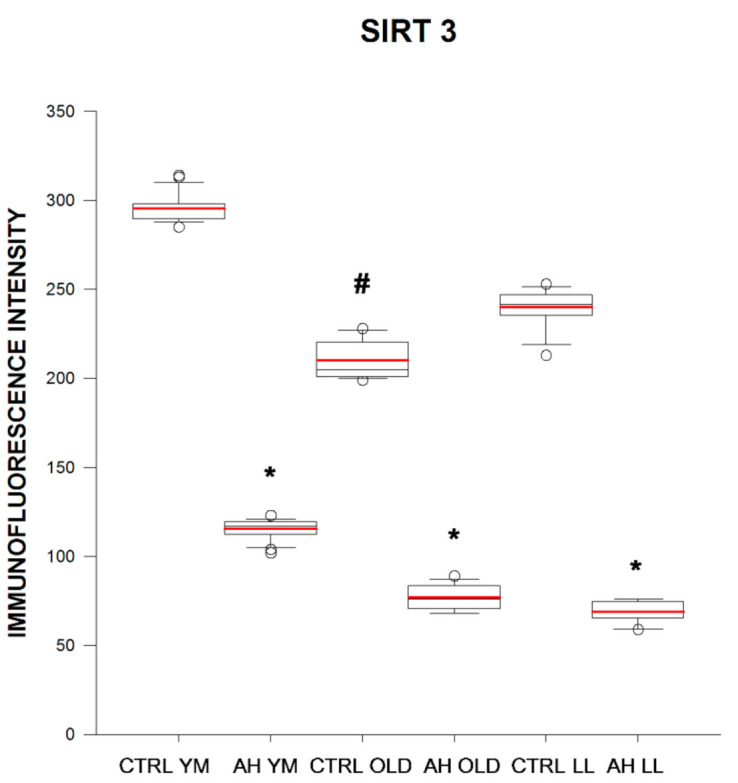
Immunofluorescence intensity (arbitrary units) of SIRT3 in buccal epithelium in healthy subjects (CTRL) and in patients with arterial hypertension (AH) of different ages (young–middle-aged YM, elderly OLD, and long-lived LL). * *p* < 0.05 as compared with the respective age-matched controls for patients with AH, **#** *p* < 0.05 as compared with the other age groups for controls and patients with AH. Box and whiskers show minimum, Q1, median (black line), mean (red line), Q3, maximum, outliers (circle).

**Figure 6:**
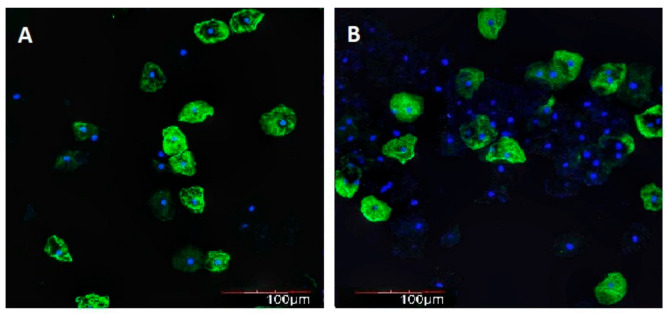
Immunofluorescence intensity of SIRT3 in buccal epithelium. 100×. Immunofluorescence staining with antibodies to SIRT3 (Alexa Fluor 488—green fluorescence). Nuclei counterstained with Hoechst 33258—blue fluorescence. (**A**) Woman, 28 years old, control, (**B**) woman, 93 years old, healthy.

SIRT3 immunofluorescence intensity in BE in elderly and long-lived controls was 1.4 and 1.3 times lower as compared with that in young/middle-aged controls, respectively. In young/middle-aged, elderly, and long-lived AH patients SIRT3 immunofluorescence intensity in BE was 2.6, 2.7, and 3.5 times lower as compared with that in age-matched controls, respectively. In elderly and long-lived AH patients, SIRT3 immunofluorescence intensity in BE was 1.5 and 1.7 times lower as compared with that in young/middle-aged AH patients, respectively (Figure 5 and Figure 6). In long-lived controls SIRT3 immunofluorescence intensity in BE was negatively correlated with subject weight (*R* = −0.615, *p* = 0.0389) and MT immunofluorescence intensity in BE was negatively correlated with systolic blood pressure values (*R* = −0.687, *p* = 0.0165).

**Figure 7:**
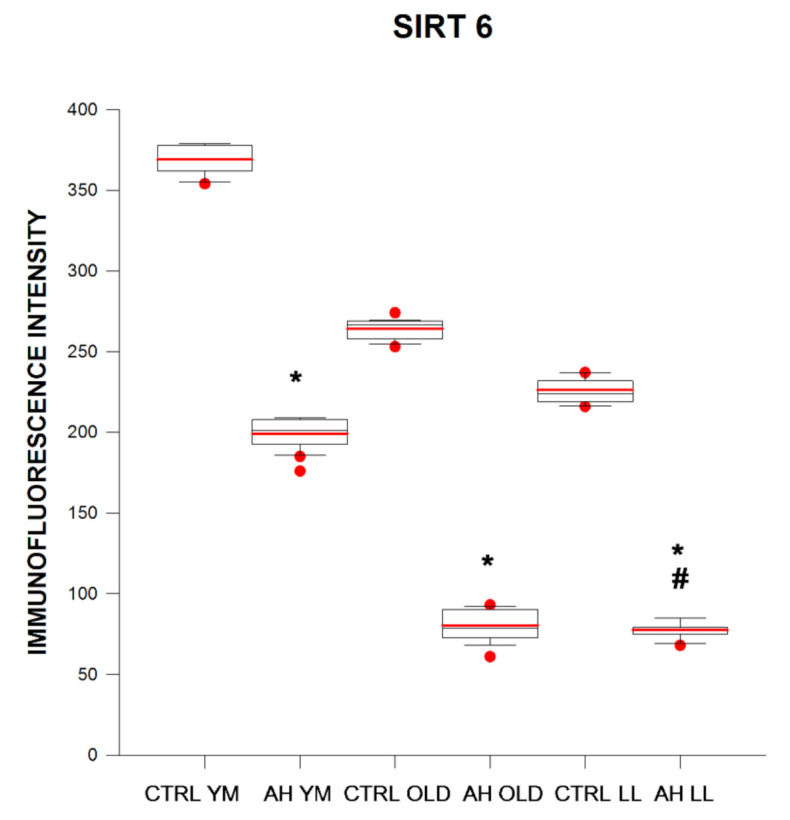
Immunofluorescence intensity (arbitrary units) of SIRT6 in buccal epithelium in healthy subjects (CTRL) and in patients with arterial hypertension (AH) of different ages (young–middle-aged YM, elderly OLD, and long-lived LL). * *p* < 0.05 as compared with the respective age-matched controls for patients with AH, **#** *p* < 0.05 as compared with the other age groups for controls and patients with AH. Box and whiskers show minimum, Q1, median (black line), mean (red line), Q3, maximum, outliers (circle).

**Figure 8:**
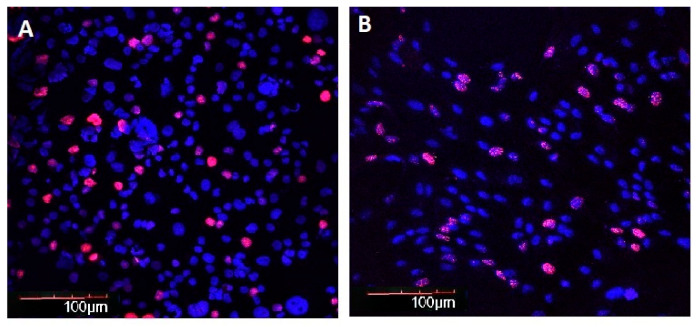
Immunofluorescence intensity of SIRT6 in buccal epithelium. 200×. Immunofluorescence staining with antibodies to SIRT6 (Alexa Fluor 567—pink fluorescence). Nuclei counterstained with Hoechst 33258—blue fluorescence. (**A**) Man, 91 years old, control, (**B**) man, 98 years old, arterial hypertension.

Considering the control groups, SIRT6 immunofluorescence intensity in BE was 1.4 and 1.6 times lower in elderly and long-lived controls when compared with young/middle-aged controls, respectively. Comparing control and AH groups, SIRT6 immunofluorescence intensity in BE was 1.89, 3.32, and 2.89 times lower in young/middle-aged, elderly, and long-lived AH patients as compared with age-matched controls, respectively. Considering the AH patients, SIRT6 immunofluorescence intensity in BE was 2.5 and 2.6 times lower in elderly and long-lived AH patients as compared with young/middle-aged AH patients, respectively (Figure 7 and Figure 8). Correlation and regression analyses showed that SIRT6 immunofluorescence intensity in BE in young/middle-aged AH patients was positively correlated with systolic blood pressure values (R = 0.585, *p* = 0.005), and the variable weight appeared to account for the ability to predict it (*p* < 0.05).

## 3. Discussion

The aging process is not uniform even taking into consideration the same age groups and tools useful for assessing the physiological age and the progress of physiological aging at an individual level are represented by biomarkers of aging. In this context, BE sampling is a non-invasive method to harvest useful material for evaluating the expression of genes and proteins as well as the synthesis of molecules involved in the aging process with and without concomitant pathologies. The collected samples can be examined with different non-imaging techniques, such as flow cytometry, and with imaging techniques, such as microscopy imaging. We used RT-PCR to evaluate SIRT1,3,6 mRNA expression and assessed MT and sirtuins in BE using a qualitative/semi-quantitative, cell-based immunofluorescence assay. The main advantage of microscopy imaging and cytometry is the possibility for attaining morphological and spatial information as well as quantification of differences, allowing comparison between different samples based on objective data.

Our study showed that in elderly and long-lived controls and AH patients, MT immunofluorescence intensity in BE decreases as compared with young/middle-aged controls as well as AH patients, respectively, in agreement with the reported age-related decrease in MT in blood [21] and excretion of its 6-sulfatoxymelatonin metabolite in urine [22].

Interestingly, in young/middle-aged AH patients MT immunofluorescence intensity in BE was lower when compared with age-matched controls, suggesting that a decrease in MT synthesis could associate with the development of AH, and this association could become even stronger with aging. This hypothesis could be supported by the fact that MT affects the functioning of the renin-angiotensin system, which is deeply involved in blood pressure regulation [8].

Besides, SIRT3 immunofluorescence intensity in BE was negatively correlated with weight in long-lived controls, and MT intensity was negatively correlated with systolic blood pressure values, whereas SIRT6 immunofluorescence intensity was positively correlated with systolic blood pressure values and was predicted by the variable weight in young/middle-aged AH patients.

MT exerts geroprotective functions in particular at the level of the vascular endothelium, preventing oxidative stress and regulating SIRTs expression [23].

In the enrolled subjects, SIRT1 gene expression and immunofluorescence intensity in BE seemed affected by the presence of AH but not by age, whereas SIRT3 and SIRT6 gene expression and immunofluorescence intensity in BE was influenced both by age and presence of AH.

Overall, our results seem in agreement with previous data suggesting the vasoprotective and geroprotective effects of SIRT1, SIRT3, and SIRT6 [15,16,17,18,19,20] and pinpointing these molecules and MT as potential therapeutic targets in the context of vasoprotection [15,16,17,18,19,20] as well as geroprotection [24,25,26].

A foremost purpose of gero-science is to exploit reliable biomarkers to foresee aging rate at the individual level. To date, no biomarker or combination of biomarkers unquestionably came out without being rigorously scrutinized.

The molecular factors considered in our study could be valuable biomarkers of aging and potential tools for therapeutic strategies and this possibility will be addressed in future studies. In this regard, our data may support the possibility to evaluate the expression of MT, SIRT1, SIRT3, and SIRT6 in BE during aging and in AH patients of different ages as additional criteria for assessing the aging process and to stratify AH patients of different ages into low, intermediate, or high expression groups with potentially different risks for disease complications.

In conclusion, some take-home messages could be suggested:

∙ BE sampling is a non-invasive procedure, yielding highly informative material for evaluating the expression of genes and proteins as well as the synthesis of molecules.
∙ No data are so far available on the role of BE sampling to assess the expression of the geroprotective molecules MT and SIRTs as biomarkers of aging and age-related diseases.
∙ Gauging the expression of MT and SIRTs in BE of subjects of different ages with and without AH using RT-PCR, immunocytochemistry, and immunofluorescence microscopy yields reliable results.
∙ It can be proposed that MT and SIRTs levels evaluation in BE samples might provide valuable markers of the aging process and age-related pathologies.

## 4. Materials and Methods

### 4.1. Buccal Epithelium Samples Collection

BE can be viewed as a border zone between the internal and external environment of the body. Collection of BE samples is a non-invasive procedure. Changes in the functional activity of buccal epithelocytes to a large extent reflect the state of local and systemic homeostasis or its disruption in pathological states and aging [27,28,29]. Therefore, BE was chosen for conducting this study. BE was obtained from 100 consecutive subjects (controls and patients with AH), who voluntarily participated to the study and were examined at the Department of Clinical Gerontology of the Saint Petersburg Institute of Bioregulation and Gerontology (Saint Petersburg, Russian Federation). All patients gave their informed written consent to participate to the study. Blood pressure was measured, and BE was sampled in the morning at the same time (10 a.m.) in all subjects. For the definition of AH the following cut-off values were considered: systolic arterial pressure >140 mmHg and diastolic arterial pressure >90 mmHg.

The enrolled subjects were divided into the following age groups: 1—young and middle-aged subjects (25–59 years, average age 45.3 ± 4.3 years, *n* = 42, 18 males and 24 females); 2—elderly subjects (60–89 year, average age 68.6 ± 3.9 year, *n* = 36, 16 males and 20 females); 3—long-lived subjects (90 years and older, average age 92.1 ± 1.7 years, *n* = 22, 5 males and 17 females). Each group was divided into two subgroups: subjects without any cardiovascular pathology and/or other somatic disease (controls) and patients with Grade 1 and Grade 2 AH. Baseline characteristics of the enrolled subjects are described in Table 2. Prior to their enrollment in the study, all patients had undergone dental examination and did not have any dental and buccal pathology. During the study, all participants received standard foods and were allowed to exert the ordinary level of physical activity. The study was conducted in late fall and early winter. BE samples were collected from the oral cavity (buccal mucosa) not earlier than 4 h after meals, following mouth rinsing with a saline solution. BE samples were taken using sterile polyester swab sticks; their test parts were then cut off and placed into single-use Eppendorf tubes with a transport medium (isotonic water saline solution with preservative).

BE was collected from three zones of the oral mucosa with the cytobrush cytological technique. To carry out liquid cytology, the cytobrush was rotated 360° in a clockwise direction five times in the same direction while maintaining gentle pressure and placed in a vial. Cell smears were prepared by the liquid-based cytology technique using the Novoprep automated system (NRS, France) [30]. In each subject, the cell smears were checked by cell count to assess quality and included in the analysis if they contained at least a median of 0.1 × 10^6^ BE cells (range 0.01 × 10^6^ to 0.2 × 10^6^ cells) per cytobrush. The selected samples were examined using RT-PCR as well as immunofluorescence analysis by confocal microscopy.

### 4.2. Real-Time Polymerase Chain Reaction (RT-PCR)

Total RNA was extracted from BE cells using the RNeasy Mini Kit (Qiagen LLC, 9259 Eton Ave Chatsworth, CA, USA). RNA extraction was performed according to the manufacturer’s protocol. The first strand coding DNA (cDNA) was synthesized by using the Revert Aid First Strand cDNA Synthesis Kit (Thermo Fisher Scientific Inc, Waltham, MA, USA), with 1000 ng of RNA per 20 µL of reaction mixture. The obtained cDNA was used as a template to define gene expression by quantitative real-time PCR (qRT-PCR) at a rate of 1 µL per 24 µL of reaction mixture. The qRT-PCR was performed with an intercalating fluorescent dye (SYBR Green I) by using the QuantiFast SYBR Green PCR Kit (Qiagen LLC, 9259 Eton Ave Chatsworth, CA, USA) and CFX96 Real-Time PCR Detection System (Bio Rad Laboratories, Hercules, CA, USA). Oligonucleotide primers were designed using the NCBI Primer-Blast web service. We used pairs of primers, one of which matched the sections of two neighboring exons. Statistical analysis of results and building of diagrams was performed automatically with CFX Manager Software v 3.1. We used GAPDH mRNA as the internal standard [31] relative to housekeeping gene expression.

### 4.3. Immunofluorescence Analysis by Confocal Microscopy

Immunostaining of BE preparations was performed according to the standard protocol, using primary antibodies to MT (1:75, Cambridge, MA, USA), SIRT1 (1:100, Cambridge, MA 02139-1517. USA), SIRT3 (1:50, Cambridge, MA 02139-1517. USA), and SIRT6 (1:100, Cambridge, MA 02139-1517. USA). Moreover, we used the following conjugated secondary antibodies: Alexa Fluor 567 (1:1000, Cambridge, MA 02139-1517. USA), Alexa Fluor 488 (1:1000, Cambridge, MA 02139-1517. USA), and Alexa Fluor 555 (1:1000, Cambridge, MA 02139-1517. USA). Cell nuclei were counterstained with Hoechst 33258 (St. Louis, MO, USA). The obtained preparations were examined with an inverted confocal laser-scanning microscope (Olympus FluoviewTM FV300/IX70, Olympus America, Melville, NY, USA), Olympus digital camera, IntelPentium 5 personal computer, and ImageJ software v. 1.48 (ImageJ, U.S. National Institutes of Health, Bethesda, Maryland, USA, imagej.nih.gov/ij/). The mean fluorescence intensities were determined as the mean intensities of the fluorescent pixels after segmentation, which was performed by assigning a threshold value (constant within each experiment) of fluorescence for every image. All images were acquired at the same microscope settings, and brightness and contrast adjustments were consistent across all samples. To increase the level of detail, the original 8-bit images in xxx.pic format were transformed into xxx.tif format, standard brightness/contrast settings were set, and the final resolution was increased to 600 pixels per inch. Analysis of images (8-bit) was carried out after their conversion to binary resolution. Five fields of view were analyzed in each case. Fluorescence intensity was calculated three times for each image, these measurements were then averaged for each sample.

### 4.4. Statistical Analysis

Statistical analysis was performed with SPSS Statistics, statistic software package for Windows v. 17.0. (SPSS Inc., Chicago, IL, USA). Sample size and statistical power: for a difference in means of 50.0 and a standard deviation of residuals of 25.0 with a number of groups of 6, a sample size of 10 subjects per group would allow a statistical power of 1.00 with an α level of 0.050. Baseline characteristics of the enrolled subjects were reported as mean ± standard deviation (SD) or frequencies and percentages for continuous and categorical variables, respectively. Normal distribution assumption was checked by means of Shapiro–Wilks and Kolmogorov–Smirnov tests. Continuous and categorical variables were evaluated using the Kruskal–Wallis one-way analysis of variance on ranks followed by all pairwise multiple comparison procedures (Dunn’s Method), Spearman rank order correlation, and multiple linear regression, as appropriate. The level at which the null hypothesis is rejected was set at the proportion 0.05.

## Figures and Tables

**Table 1:** Expression of SIRT1,3,6 genes in buccal epithelium (BE) in control subjects and arterial hypertension (AH) patients of different ages.

Age Group	SIRT1		SIRT3		SIRT6	
	Control	AH	Control	AH	Control	AH
25–59 years old	1.12 ± 0.24	0.67 ± 0.11 *	1.55 ± 0.27	0.50 ± 0.07 *	2.04 ± 0.30	0.90 ± 0.11 *
60–89 years old	0.90 ± 0.20	0.50 ± 0.10 *	0.81 ± 0.19 **	0.63 ± 0.10 *	1.01 ± 0.17 **	0.31 ± 0.06 *^,†^
≥90 years old	0.87 ± 0.18	0.52 ± 0.12 *	0.86 ± 0.20 **	0.65 ± 0.11 *	0.96 ± 0.15 **	0.35 ± 0.07 *^,†^

* *p* < 0.01 as compared with the respective age-matched control. ** *p* < 0.01 as compared with 25–59 years old controls. ^†^ *p* < 0.01 as compared with 25–59 year old AH patients. All numerical values are indicated as mean ± SD. Gene expression data are referred as relative to the housekeeping gene expression.

**Table 2:** Baseline characteristics of the enrolled subjects.

Group	Age (Years)	Gender (*n*) (% of Female Subjects)	Height (cm)	Weight (kg)	Systolic Blood Pressure (mmHg)	Diastolic Blood Pressure (mmHg)
Subjects without arterial hypertension (*n* = 50) (50%)						
25–59 years old	43.6 ± 5.5	10 males, 11 females (52.3% females)	178.7 ± 5.5	83.4 ± 13.3	110.1 ± 5.3	76.0 ± 2.2
60–89 years old	70.9 ± 7.3	10 males, 8 females (44.4% females)	172.2 ± 5.1	86.5 ± 10.1	107.2 ± 2.6	78.1 ± 2.5
≥90 years old	89.9 ± 1.2	2 males, 9 females (81.8% females)	174.0 ± 6.5	87.1 ± 10.4	113.4 ± 4.6	74.2 ± 3.6
Subjects with arterial hypertension (*n* = 50) (50%)						
25–59 years old	47.0 ± 6.1	8 males, 13 females (61.9% females)	176.3 ± 9.06	89.6 ± 7.8	142.3 ± 3.1 *	89.4 ± 4.0 *
60–89 years old	66.4 ± 4.1	6 males, 12 females (66.7% females)	170.1 ± 5.3	94.1 ± 3.7	150.0 ± 3.04 *	92.0 ± 3.8 *
≥90 years old	95.1 ± 1.76	3 males, 8 females (72.7% females)	172.09 ± 7.9	95.2 ± 5.1	163.0 ± 2.5 *	96.0 ± 2.02 *

* *p* < 0.05 in comparison with the subjects of the same age group but without arterial hypertension. All numerical values are indicated as mean ± SD.
